# APE2 Is a General Regulator of the ATR-Chk1 DNA Damage Response Pathway to Maintain Genome Integrity in Pancreatic Cancer Cells

**DOI:** 10.3389/fcell.2021.738502

**Published:** 2021-11-02

**Authors:** Md Akram Hossain, Yunfeng Lin, Garrett Driscoll, Jia Li, Anne McMahon, Joshua Matos, Haichao Zhao, Daisuke Tsuchimoto, Yusaku Nakabeppu, Jianjun Zhao, Shan Yan

**Affiliations:** ^1^Department of Biological Sciences, University of North Carolina at Charlotte, Charlotte, NC, United States; ^2^Division of Neurofunctional Genomics, Department of Immunobiology and Neuroscience, Medical Institute of Bioregulation, Kyushu University, Fukuoka, Japan; ^3^Department of Cancer Biology, Lerner Research Institute, Cleveland Clinic, Cleveland, OH, United States

**Keywords:** ATR-Chk1, DNA damage response, DNA double-strand breaks, DNA single- strand breaks, genome integrity, APE2

## Abstract

The maintenance of genome integrity and fidelity is vital for the proper function and survival of all organisms. Recent studies have revealed that APE2 is required to activate an ATR-Chk1 DNA damage response (DDR) pathway in response to oxidative stress and a defined DNA single-strand break (SSB) in *Xenopus laevis* egg extracts. However, it remains unclear whether APE2 is a general regulator of the DDR pathway in mammalian cells. Here, we provide evidence using human pancreatic cancer cells that APE2 is essential for ATR DDR pathway activation in response to different stressful conditions including oxidative stress, DNA replication stress, and DNA double-strand breaks. Fluorescence microscopy analysis shows that APE2-knockdown (KD) leads to enhanced γH2AX foci and increased micronuclei formation. In addition, we identified a small molecule compound Celastrol as an APE2 inhibitor that specifically compromises the binding of APE2 but not RPA to ssDNA and 3′-5′ exonuclease activity of APE2 but not APE1. The impairment of ATR-Chk1 DDR pathway by Celastrol in *Xenopus* egg extracts and human pancreatic cancer cells highlights the physiological significance of Celastrol in the regulation of APE2 functionalities in genome integrity. Notably, cell viability assays demonstrate that APE2-KD or Celastrol sensitizes pancreatic cancer cells to chemotherapy drugs. Overall, we propose APE2 as a general regulator for the DDR pathway in genome integrity maintenance.

## Introduction

Cells undergo continuous bombardments of exogenous and endogenous factors that can lead to genomic instability. It is critical for a cell to maintain genome integrity and fidelity for proper cellular function and survival in stress conditions. This task is daunting due to constant insults on the DNA by genotoxic agents, nucleotide mis-incorporation or deprivation during DNA replication, and the intrinsic biochemical instability of the DNA itself (Lindahl, 1993). Both exogenous and endogenous sources can result in DNA replication stress and/or DNA lesions that include DNA double-strand breaks (DSB), DNA single-strand breaks (SSBs), and oxidative DNA damage (Ciccia and Elledge, 2010; Yan et al., 2014; Tubbs and Nussenzweig, 2017). Although cells have evolved several different DNA repair pathways to resolve DNA lesions, deficiency in DNA repair pathways or failure to resolve replication stress may result in blockage or collapse of replication and transcription machinery, leading to cellular cytotoxicity, mutagenesis, and/or cell death (Friedberg, 2003; Yan et al., 2014). In humans, DNA lesions are involved in numerous genetically inherited disorders, aging and carcinogenesis (Friedberg, 2003; Tubbs and Nussenzweig, 2017). In response to DNA damage, cells have also evolved the DNA damage response (DDR) pathways to coordinate DNA repair, transcription activation, cell cycle progression, and cell death (Jackson and Bartek, 2009; Ciccia and Elledge, 2010). ATM (Ataxia telangiectasia mutated) and ATR (ATM and Rad3-related) kinases are the key regulators in DDR pathways. Whereas ATM-mediated DDR pathway is primarily activated in response to DSBs, ATR-mediated DDR pathway is triggered by several types of stressful conditions, including DNA replication stress, oxidative stress, SSBs, and DSBs (Cimprich and Cortez, 2008; Marechal and Zou, 2013; Paull, 2015). The ATR DDR pathway is critical for duplicating DNA under stressful conditions (Saldivar et al., 2017), and ATR inhibitors as either monotherapy or combination therapy have been in different phases of clinical trials of cancer patients (Karnitz and Zou, 2015; Bradbury et al., 2020).

Depending on the nature and context of DNA damage or replication stress, the ATR DDR pathway is activated by different regulatory mechanisms. It has been proposed that single-strand DNA (ssDNA) coated with RPA (i.e., RPA-ssDNA) together with a 5′-ssDNA/dsDNA junction may serve as a platform to recruit ATR DDR complexes including ATR, ATRIP, TopBP1, and the Rad9-Rad1-Hus1 (9-1-1) complex for ATR activation (Cimprich and Cortez, 2008; Marechal and Zou, 2015). In DNA replication stress, stalled DNA replication forks induced by aphidicolin or gemcitabine (GEM) uncouple helicase and DNA polymerases, generating RPA-ssDNA for ATR activation (Byun et al., 2005; Yan and Michael, 2009; Fredebohm et al., 2013). In response to DSBs induced by γ-radiation, Topoisomerase I inhibitor Camptothecin (CPT), and Topoisomerase II inhibitor Etoposide (ETO), ATR can also be activated by ssDNA derived from bidirectional DSB end resection by different endonucleases and exonucleases such as Mre11 and Exo1 (Shiotani and Zou, 2009; Symington, 2014; Daley et al., 2015). Oxidative DNA damage induced by hydrogen peroxide (H_2_O_2_) also activates ATR DDR pathway by generating ssDNA at oxidative damage sites (Willis et al., 2013; Wallace et al., 2017). Recent studies have demonstrated that defined SSB structures can activate the ATR DDR pathway via a distinct 3′-5′ end resection mechanism that generates necessary short ssDNA (Hossain et al., 2018; Lin et al., 2018).

APE2 (Apurinic/apyrimidinic endonuclease-2, also known as APEX2 or APN2) is an evolutionarily conserved protein with strong 3′-phosphodiesterase and 3′-5′ exonuclease activities but weak AP endonuclease activity and has been implicated in genome and epigenome integrity maintenance (Burkovics et al., 2009; Chaudhari et al., 2021; Lin et al., 2021). Prior studies using different model systems have shown that APE2 plays crucial roles in DNA repair pathways including the base excision repair (BER) pathway, SSB repair pathway, DSB generation and DSB repair pathway, DDR pathways including the ATR-Chk1 DDR pathway and p53-dependent DDR pathway, immune responses including immunoglobulin somatic hypermutation (SHM) and class switch recombination (CSR), and active DNA demethylation (Unk et al., 2001; Burkovics et al., 2006, 2009; Guikema et al., 2007; Dan et al., 2008; Sabouri et al., 2009; Willis et al., 2013; Li et al., 2018; Lin et al., 2018, 2020; Cupello et al., 2019; Yan, 2019; Alvarez-Quilon et al., 2020). Furthermore, APE2 has been implicated in development and growth as well as cancer etiology. A prior study has shown that APE2-knock out (KO) mice are viable but display growth retardation (Ide et al., 2004). Accumulating evidence has shown genomic alterations and abnormal expression of APE2 expression in multiple cancer tissues, including pancreatic cancer and multiple myeloma (MM), and APE2 is proposed to function as an oncogene in liver cancer (Kumar et al., 2018; Jensen et al., 2020; Zheng et al., 2020). Although the underlying molecular mechanism remains to be determined, recent genetic screens identified APE2 as a synthetic lethal target in BRCA1- or BRCA2-deficient human colon cancer cell line DLD-1, human ovarian cancer cell line PEO1, or engineered human epithelial cell line RPE1-hTERT under unperturbed conditions (Mengwasser et al., 2019; Alvarez-Quilon et al., 2020). It has been demonstrated in recent series of studies using *Xenopus* egg extracts that APE2 is critical for the ATR-Chk1 DDR pathway in response to oxidative DNA damage and defined SSB structures (Willis et al., 2013; Wallace et al., 2017; Lin et al., 2018). Mechanistically, APE2 is recruited to oxidative stress-derived SSB sites or defined SSB structures for a distinct 3′-5′ SSB end resection via its 3′-5′ exonuclease activity, leading to RPA-ssDNA, assembly of the ATR DDR complex including ATR, ATRIP, TopBP1, and the 9-1-1 complex, and activation of the ATR DDR pathway (Willis et al., 2013; Wallace et al., 2017; Hossain et al., 2018; Lin et al., 2018). Moreover, APE2 recruitment and activation require its interaction with ssDNA via its C-terminal Zf-GRF motif and two modes of association with PCNA via its Zf-GRF motif and PCNA-Interacting Protein box (PIP) (Wallace et al., 2017; Lin et al., 2018). APE2 directly associates with and brings Chk1 to the activated ATR for phosphorylation (Willis et al., 2013). However, it remains largely unknown whether and how APE2 regulates the ATR DDR pathway in response to different stressful conditions in mammalian cells.

With a ∼9% 5-year survival rate for all stages combined, pancreatic cancer ranks the fourth most common form of cancer-related deaths in the United States, with nearly 57,600 estimated new cases and over 55,000 estimated deaths in 2020 (Siegel et al., 2020). Although GEM has been the standard treatment of pancreatic cancer, the clinical effect of GEM monotherapy remains limited such as low overall survival months and efficacy (Merl et al., 2010). In contrast, new therapy regimen such as a modified FOLFIRINOX regimen (a combination of fluorouracil, leucovorin, irinotecan, and oxaliplatin) as an adjuvant therapy after surgical resection of pancreatic cancer is still developing (Conroy et al., 2018). A combination of GEM with radiotherapy or other chemotherapy drugs such as ATR inhibitor AZD6738 shows great promise in pancreatic cancer regression (Wallez et al., 2018). Because targeting ATR has emerged as a new area of research for cancer treatment (Fokas et al., 2012; Karnitz and Zou, 2015; Bradbury et al., 2020), it is reasonable to investigate and explore innovative therapy via targeting the ATR-Chk1 DDR pathway’s regulatory mechanisms to increase efficacy and/or reduce the toxicity of chemotherapy drugs in pancreatic cancer treatment.

This study provides evidence using pancreatic cancer cell lines that activation of the ATR-Chk1 DDR pathway induced by hydrogen peroxide (H_2_O_2_), GEM, CPT, and ETO is compromised when APE2 is down-regulated via siRNA. Furthermore, siRNA-mediated APE2-knockdown (KD) leads to a higher percentage of γH2AX-positive cells and micronuclei-positive cells. These results suggest that APE2 is a general regulator of the ATR-Chk1 DDR pathway to maintain genome integrity. In addition, we found that Celastrol, a natural compound derived from thunder god vine *Tripterygium wilfordii* (Lu et al., 2020), impaired APE2 interaction with ssDNA and APE2 3′-5′ exonuclease activity *in vitro* and also compromised the defined SSB-induced ATR-Chk1 DDR pathway in *Xenopus* egg extracts. Notably, the ATR-Chk1 DDR pathway activation induced by H_2_O_2_, GEM, CPT, and ETO in pancreatic cancer cells was compromised by the addition of Celastrol. Cell viability assays demonstrated that APE2 suppression via siRNA-mediated KD or the addition of Celastrol sensitized pancreatic cancers to chemotherapy drugs. Our evidence suggests that APE2 regulates the ATR DDR pathway in pancreatic cancer cells and that targeting the novel function of APE2 in ATR DDR may open a new avenue for future therapeutics in pancreatic cancers.

## Materials and Methods

### Cell Culture, Treatments and Cell Lysate Preparation

PANC1 and MiaPaCa2 cells were purchased from ATCC (Cat#CRL-1469 and CRL-1420) and cultured in complete media [DMEM (Corning) supplemented with 10% FBS (Atlanta Biologicals) and 1% penicillin/streptomycin (Gibco)] for PANC1 or completed media with 2.5% Horse Serum (Sigma) for MiaPaCa2, respectively. Cells were treated with H_2_O_2_ (Sigma Cat#HX0635), Gemcitabine (GEM, Sigma Cat#G6423) Camptothecin (CPT, Calbiochem Cat#208925), Etoposide (ETO, Calbiochem Cat#341205), VE-822 (Selleckchem Cat#S7102), KU55933 (EMD Millipore Cat#118500), or Celastrol (Sigma Cat#219465) to the final concentrations and incubated for the times as indicated in the individual experiments. GEM, CTP, ETO, VE-822, KU55933, and Celastrol were dissolved in DMSO and stored at −20°C.

Briefly, cells were washed with phosphate-buffered saline, PBS (Gibco Cat#10010023) and trypsinized (Corning Cat#25-053-CI). The cells were collected by centrifugation and resuspended in ice-cold PBS followed by centrifugation. Cultured cells were lysed with in lysis buffer (20 mM Tris–HCL pH 8.0, 150 mM NaCl, 2 mM EDTA, 0.5% Non-idet P-40, 0.5 mM Na_3_V0_4_, 5 mM NaF, 5 μg/mL of Aprotinin, and 10 μg/mL of Leupeptin). Lysates were centrifugated at 13,000 rpm for 30 min at 4°C. The supernatants were transferred into fresh tubes for measuring protein concentrations via Bradford assays (BIO-RAD Cat#5000205) and subsequent immunoblotting analysis.

### Transfection and siRNA-Mediated APE2-KD Assays

**FIGURE 1:**
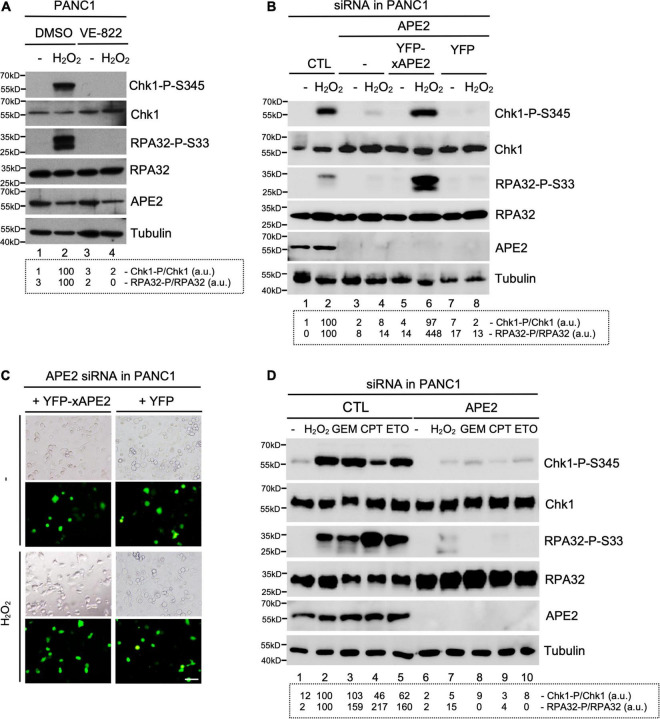
APE2 is important for the activation of the ATR-Chk1 DDR pathway in pancreatic cancer cells. **(A)** After 1-h pretreatment of VE-822 (5 μM), PANC1 cells were added with or without H_2_O_2_ (1 mM) for 4 h. Cell lysates were examined via immunoblotting analysis as indicated. **(B)** Transfecting YFP-xAPE2 but not YFP can rescue the Chk1 and RPA32 phosphorylation induced by H_2_O_2_ in APE2-KD PANC1 cells. The ATR-Chk1 DDR pathway analysis of cell lysates from different samples were examined via immunoblotting as indicated. **(C)** Fluorescence microscopy analysis shows that YFP-xAPE2 or YFP was expressed similarly in APE2-KD PANC1 cells with or without treatment of H_2_O_2_. Scale bar, 100 μm. **(D)** The ATR DDR signaling in cell lysates of PANC1 cells with control (CTL) or APE2 siRNAs after treatment of various DNA damaging condition was examined via immunoblotting analysis as indicated. Cells were treated with H_2_O_2_ (1 mM), GEM (50 μM), CPT (5 μM), or ETO (50 μM) for 4 h. Quantifications of Chk1-P/Chk1 (a.u.) and RPA32-P/RPA32 (a.u.) were shown in a dashed box under the immunoblots. The immunoblotting analysis results are representative from two independent experiments.

For siRNA experiments, APEX2 siRNA (Dharmacon-HorizonDiscovery ON-TARGETplus Human APE2 siRNA Cat#L-013730-01-0005) or control siRNA (Dharmacon-HorizonDiscovery ON-TARGETplus non-targeting siRNA Cat#D-001810-01-05) was mixed with Lipofectamine^*R*^ RNAiMAX (Thermo Fisher Scientific Cat#13778100) in Opti-MEM I Reduced Serum Medium (Gibco Cat#31985070) and incubated for 3–5 days according to the manufacture’s protocol. The target sequences of the Dharmacon APE2 siRNA include 5′-GAGCCAUGUGAUGCGUA-3′, 5′-CAACAAUCAAACCCGGGUA-3′, 5′-GGACGAGCUGGAUG CGGAU-3′, and 5′-GAGAAGGAGUUACGGACCU-3′, whereas the non-targeting siRNA sequence is 5′- UGGUUUACAUGUCGACUAA-3′. For the rescue experiments in Figure 1B and Supplementary Figure 1A, after siRNA-mediated APE2-KD, transfecting control plasmid pcDNA3-YFP (Addgene Cat#13033) or pcDNA3-YFP-xAPE2 with Lipofectamine 2000 (Thermo Fisher Scientific Cat#116680019) in Opti-MEM I Reduced Serum Medium. After different treatment and incubation, cells were imaged via fluorescence microscopy to ensure YFP or YFP-xAPE2 was expressed in cells.

### Immunofluorescence Analysis

Cells were fixed in 3% formaldehyde solution for 15 min at room temperature and permeabilized with 2% Triton-X 100. Cells were then incubated with antibodies again γH2AX (EMD Millipore Cat#05-636-AF488, anti-phospho Histone H2AX Ser139-Alexa Fluor 488 conjugate) or APE2 (GeneTex Cat#GTX80642) overnight at 4°C. For APE2 experiment, goat anti-rabbit IgG H&L-conjugated with Alexa Fluor 594 (Abcam Cat#ab150080) was probed as the secondary antibodies. Then cells were mounted with ProLong Gold Antifade Mountant with DAPI (Invitrogen Cat#36941) before immunofluorescence imaging by confocal laser scanning microscope (Olympus FluoView FV1000) or upright fluorescence microscope (Leica DM6 B) analyses.

### Cell Viability Assays

**FIGURE 2:**
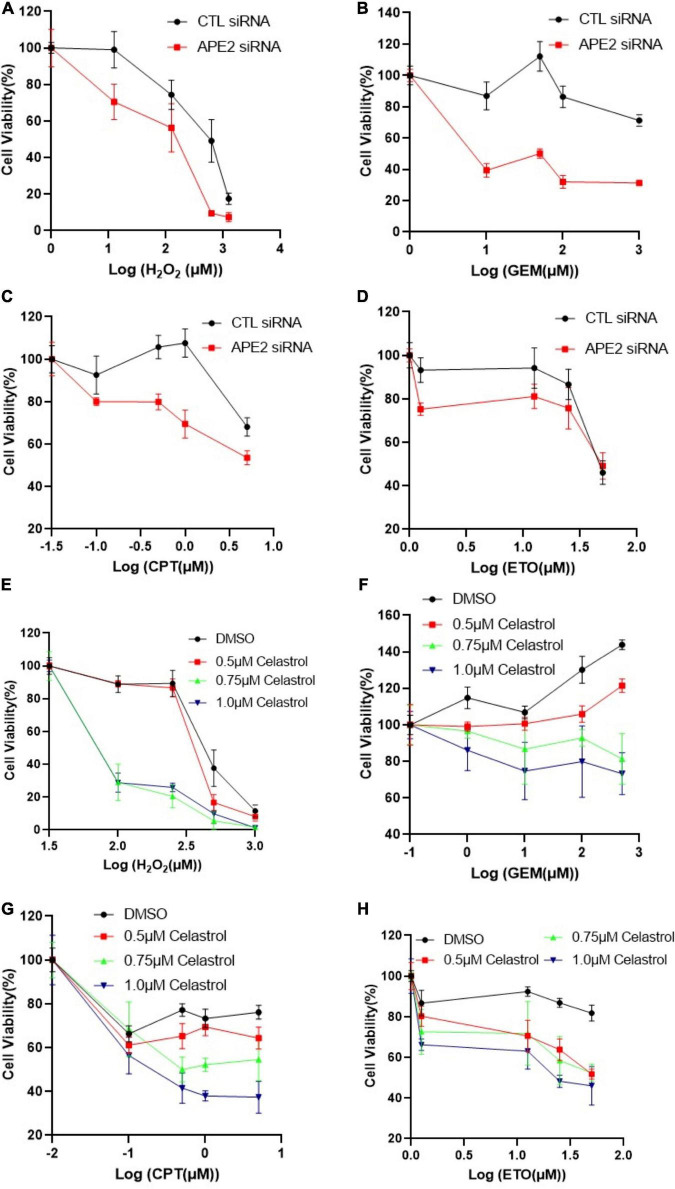
APE2 suppression or Celastrol sensitizes PANC1 cells to chemotherapy drugs. **(A–D)** Cell viability assays show that APE2-KD PANC1 cells are more vulnerable to stress conditions [H_2_O_2_ **(A)**, GEM **(B)**, CPT **(C)**, or ETO **(D)**] than Control (CTL) siRNA transfected cells. **(E–H)** Cell viability assays demonstrate that Celastrol (0.5, 0.75, or 1 μM) sensitizes PANC1 cells to H_2_O_2_ **(E)**, GEM **(F)**, CPT **(G)**, or ETO **(H)**. After different treatment as indicated, cells were incubated for 72 h before cell viability assays via CellTiter-GLO method **(B,E)** or MTT method (other experiments).

Cell viability assay was carried out to assess percentage of viable cells via CellTiter-GLO 2.0 assays (in experiments in Figures 2B,E, and Supplementary Figure 4G) or MTT (Thiazolyl blue tetrazolium bromide) assays (other cell viability assay experiments). We performed both of the techniques and got similar results by analyzing the raw data of absorbance values (in MTT) or luminescence values (in CellTiter-GLO 2.0) using Microsoft Excel Spreadsheet. Pancreatic cells were seeded at 3,000 cells/well in transparent 96-well plates for MTT assays or Opaque 96-well plates for CellTitreGLO-2.0 assays. After different treatment as indicated in different experiments, cells are incubated for 72 h before cell viability assays. For MTT assays, each well with cells (in 100 μl) was added 20 μl of MTT reagent (5 mg/mL, Acros Organics Cat#158992500) and incubated at 37°C for 3.5 h. After cell medium was removed, 150 μl of MTT solvent (VWR Chemicals, Isopropyl ethanol and 37 M Hydrochloric acid) was added to each well for a 10-min incubation with rocking and a subsequent 5-min incubation without rocking. For CellTiter-GLO assays, 100 μl of CellTiter-GLO 2.0 reagent (Promega Cat#G9241) was added to each well with cells (100 μl) followed by incubation at room temperature for 10 min. MTT (absorbance, abs) and CellTiter-GLO 2.0 (luminescence, lum) values were determined by SpectraMAX iD5 Multiplate Reader (Thermo Fisher Scientific). The MTT/CellTiter-GLO 2.0 values were calculated based on Percentage (%) = [100 × (sample abs/lum)/(control abs/lum)]. MTT/CellTiter-GLO 2.0 assay analyses using Microsoft Excel and GraphPad PRISM software were performed in triplicates (*n* = 3). Data are presented as mean ± SD for the error bars and normalized with no treatment group.

### Recombinant DNA, Plasmid DNA, FAM-Labeled DNA Structures, and Recombinant Proteins

**FIGURE 3:**
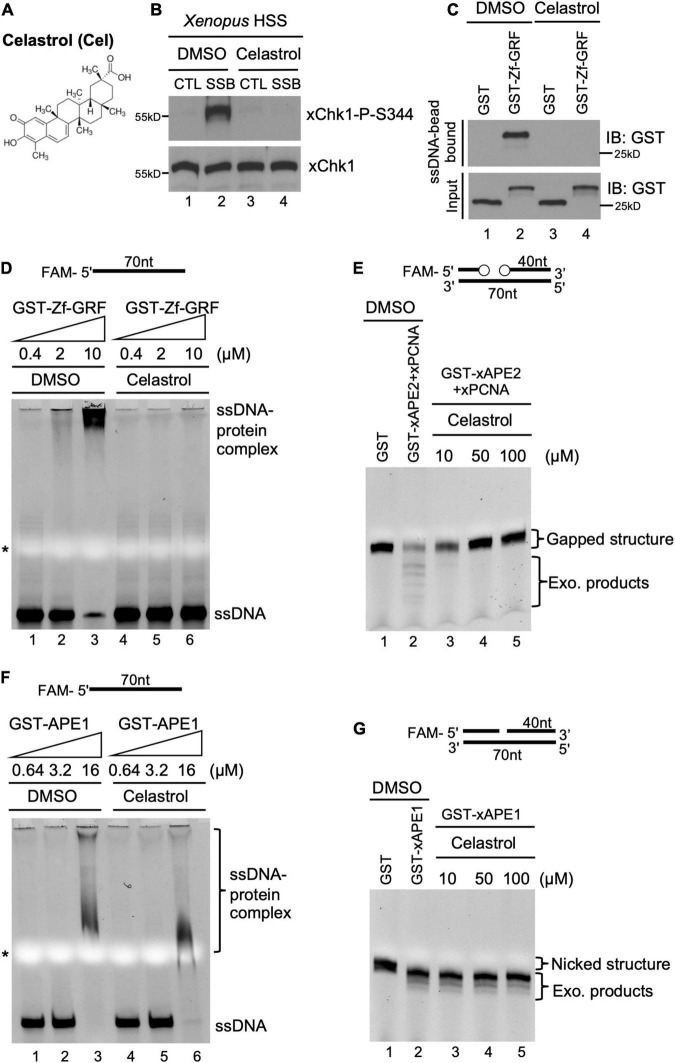
A small molecule inhibitor Celastrol impairs APE2 function in the SSB-induced ATR DDR pathway in the *Xenopus* system. **(A)** Chemical structure of a small molecule inhibitor compound Celastrol. **(B)** Celastrol (1 mM) compromises Chk1 phosphorylation induced by SSB plasmid but not CTL plasmid in the *Xenopus* HSS system via immunoblotting analysis. **(C)** The binding of recombinant GST-Zf-GRF but not GST to Dynabead coupled with ssDNA was impaired by Celastrol in GST-pulldown assays. **(D)** The binding of GST-Zf-GRF to ssDNA was impaired by Celastrol in EMSA assays. **(E)** The PCNA-stimulated 3′-5′ exonuclease activity of xAPE2 on a gapped DNA structure *in vitro* was inhibited by Celastrol in TBE-Urea gel electrophoresis. **(F)** EMSA assays show that Celastrol almost had no effect on the binding of GST-xAPE1 to 70nt-ssDNA *in vitro*. **(G)** Celastrol was dispensable for the 3′-5′ exonuclease activity of GST-xAPE1 on a nicked DNA structure in TBE-Urea gel electrophoresis *in vitro*.

pcDNA3-YFP was a gift from Doug Golenbock (Addgene plasmid Cat#13033; http://n2t.net/addgene:13033; RRID:Addgene_13033). Recombinant pcDNA3-YFP-xAPE2 was prepared by subcloning the full-length of xAPE2 into pcDNA3-YFP at EcoR1 and *Xho*I sites. Briefly, the coding region of xAPE2 was amplified by PCR with a forward oligo (5′-GGGGGGAATTCATGAAGATTGTGAGCTGGAACATCAAT G-3′) and a reverse oligo (5′-GGGGGCTCGAGGTCCTCA CATCCAGCTTTTTTGGTGAG-3′). Purified PCR product and pCDNA3-YFP were catalyzed by *Eco*RI (New England Biolabs Cat#R3101) and *Xho*I (New England Biolabs Cat#R0146) and ligated together by T4 DNA ligase (New England Biolabs Cat#m0202). After transformation into DH5alpha *E. coli*, plasmids were prepared via QIAprep Spin Miniprep Kit (QIAGEN Cat#27106) following vendor’s protocol. In addition, the control (CTL) plasmid, SSB plasmid, FAM-labeled 70-nt ssDNA, and FAM-labeled 70-bp dsDNA with a gap or nicked structure in Figure 3 were described previously (Lin et al., 2018, 2019, 2020). The pET32a-hAPE2 was described previously (Tsuchimoto et al., 2001). The expression and purification of recombinant protein GST-xAPE1, GST-Zf-GRF, GST-xAPE2, His-tagged xPCNA, and His-tagged RPA complex in Figure 3 and Supplementary Figure 3 has been described recently (Willis et al., 2013; Acevedo et al., 2016; Lin et al., 2018; Lin et al., 2020). The His-tagged human APE2 recombinant protein was expressed and purified as described previously (Tsuchimoto et al., 2001).

### Electrophoretic Mobility Shift Assays

For the Electrophoretic mobility shift assays (EMSA) assays in Figures 3D,F, similar method has been described previously (Lin et al., 2018, 2020). Briefly, different concentrations of purified recombinant proteins were incubated with 0.15 μM of FAM-labeled 70-nt ssDNA in EMSA Reaction Buffer (10 mM Tris pH 8.0, 50 mM NaCl, 0.2 mM TCEP, 5% glycerol) with or without Celastrol for 3 h at 4°C. Reaction samples were resolved on TBE native gel and virtualized on a BioRad imager.

### Protein-DNA Interaction Assays and *in vitro* Exonuclease Assay

**FIGURE 4:**
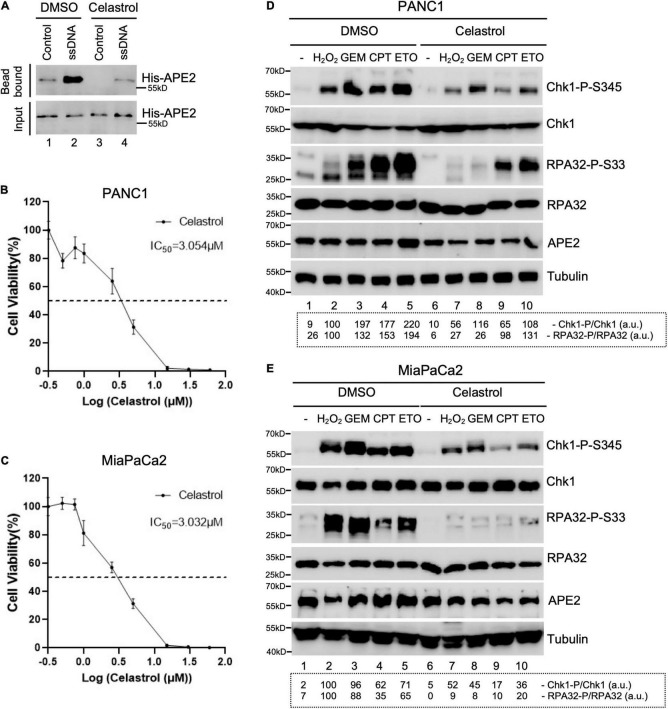
Celastrol compromises the ATR-Chk1 DDR pathway activation in pancreatic cancer cells. **(A)** The binding of recombinant His-tagged human APE2 protein to beads coupled with ssDNA was compromised by the addition of Celastrol (100 ng/μl). **(B,C)** Cell viability assays show the toxicity of Celastrol in PANC1 **(B)** or MiaPaCa2 **(C)** cells after 3-day treatment. **(D,E)** PANC1 **(D)** or MiaPaCa2 **(E)** cells were pretreated with Celastrol (2.5 μM) for 1 h followed by 4-h treatment of H_2_O_2_ (1 mM), GEM (50 μM), CPT (5 μM), or ETO (50 μM), respectively. Cell lysates were then extracted and examined via immunoblotting analysis as indicated. Quantifications of Chk1-P/Chk1 (a.u.) and RPA32-P/RPA32 (a.u.) were shown in a dashed box under the immunoblots. The immunoblotting analysis results are representative from two independent experiments.

A similar method for the ssDNA-bead binding assays in Figures 3C, 4A was described recently (Lin et al., 2018). The Input and Bead-bound fractions were analyzed via immunoblotting analysis as indicated. Similar methods for the *in vitro* exonuclease assay of APE2 (Figure 3E) and APE1 (Figure 3G) were described previously (Lin et al., 2018). Briefly, FAM-labeled 70-bp dsDNA with a gap structure (0.5 μM) was incubated with purified recombinant GST or GST-APE2/His-PCNA with different concentrations of Celastrol in Exonuclease Assay Buffer (50 mM HEPES, pH 7.5, 20 mM KCl, 10 mM MgCl_2_, 2 mM DTT). Similarly, FAM-labeled 70-bp dsDNA with a nick structure (0.5 μM) was incubated with purified recombinant GST or GST-APE1 (4 μM) in Exonuclease Assay Buffer. After 1 h-incubation at 37°C, exonuclease assay reactions were quenched with equal volume of TBE-Urea Sample Buffer and denatured at 95°C for 5 min. Samples were resolved on TBE–urea PAGE and imaged on a BioRad imager.

### Experimental Procedures for *Xenopus* Egg Extracts and the SSB-Induced DDR Pathway Assays

The preparation of *Xenopus* HSS and the similar setup of SSB-induced DDR pathway assays for Figure 3B were recently described (Willis et al., 2012; Yan and Willis, 2013; DeStephanis et al., 2015; Cupello et al., 2016; Lin et al., 2018, 2019). Briefly, SSB plasmid or control plasmid was added to the HSS to a final concentration of 75 ng/μL and incubated for 30 min at room temperature. Then the samples were examined via immunoblotting analysis.

### Immunoblotting Analysis and Antibodies

Immunoblotting analysis of cell lysates or *Xenopus* egg extracts was carried out similarly as we described previously (Willis et al., 2013; Wallace et al., 2017; Lin et al., 2018, 2020). Primary antibodies against Chk1 (Santa Cruz Biotechnology Cat#sc-8408), Chk1 phosphorylation Ser345 (Cell Signaling Technology Cat#133D3), RPA32 (Thermo Fisher Scientific Cat#MA1-26418), RPA32 phosphorylation Ser33 (Bethyl Laboratories Cat#A300-246A), and Tubulin (Santa Cruz Biotechnology Cat#sc-8035) were purchased from various vendors. Anti-human APE2 antibodies were prepared as described previously (Tsuchimoto et al., 2001).

### Quantification and Statistical Analyses

**FIGURE 5:**
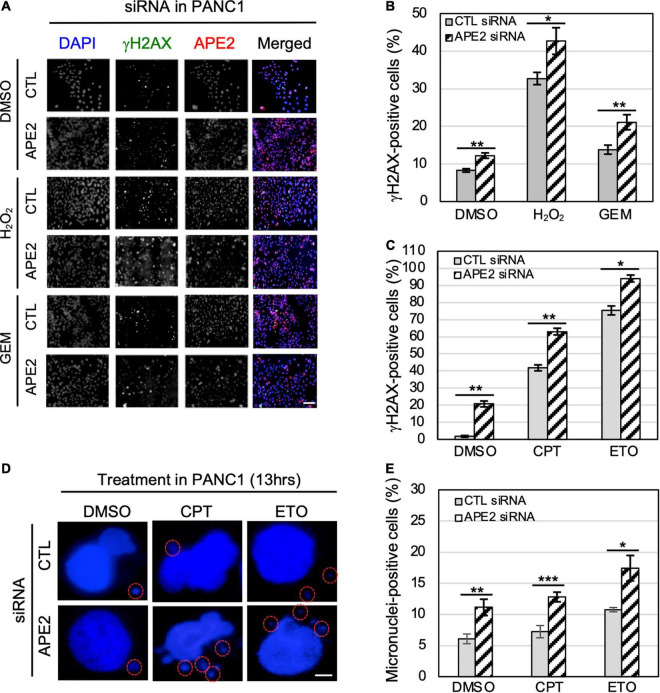
APE2-KD induces substantially more γH2AX foci and micronuclei under normal or stress conditions in PANC1 cells. **(A)** Immunofluorescence microscopy analysis shows γH2AX and APE2 foci after DMSO or treatment of H_2_O_2_ (1 mM for 5 h) or GEM (100 μM for 5 h) in PANC1 cells with CTL or APE2 siRNA in a slide view. Scale bar, 100 μm. **(B,C)** Percentage of γH2AX-positive PANC1 cells after treatment of H_2_O_2_/GEM or CPT/ETO. **(D)** Microscopy analysis shows micronuclei (circled with red) after DAPI staining in PANC1 cells after treatment of CPT (1 μM for 13 h) or ETO (20 μM for 13 h) with CTL or APE2 siRNA. Scale bar, 5 μm. **(E)** Percentage of micronuclei-positive PANC1 cells after treatment of CPT or ETO with CTL or APE2 siRNA was quantified. (B,C,E)* indicates *p* < 0.05; ** indicates *p* < 0.01; *** indicates *p* < 0.001; *n* = 3.

Intensity of immunoblotting bands such as Chk1-P-S345, Chk1, RPA32-P-S33, and RPA32 was quantified using Image J in Figures 1, 4 and Supplementary Figure 1. Chk1-P/Chk1 (a.u. indicates arbitrary units) and RPA32-P/RPA32 (a.u.) were calculated when intensity of Chk1-P-S345 or RPA32-P-S33 is normalized to that of Chk1 or RPA32, respectively. Chk1-P/Chk1 (a.u.) and RPA32-P/RPA32 (a.u.) after treatment of hydrogen peroxide were set as 100 a.u. The quantification of γH2AX-positive cells in Figures 5B,C, Supplementary Figures 2B,D was carried out by eye scoring from three different images for the average percentages and standard deviations. GraphPad PRISM 8 statistical analysis software was used to perform statistical analysis of in Figures 5B,C,E, Supplementary Figures 1D,E,2B,D,F. Data were presented as mean ± SD from three experiments. A paired two-sided *t*-test was conducted to determine significance of difference. *p* < 0.05 is considered significant and *p* < 0.01 is considered highly significant.

## Results

### APE2 Is Important for the ATR-Chk1 DDR Pathway in Different Stressful Conditions in Pancreatic Cancer Cells

Our series of studies using *Xenopus* egg extracts have demonstrated that APE2 is important for the ATR DDR pathway in oxidative stress (Willis et al., 2013; Wallace et al., 2017). To determine the role of APE2 in the ATR DDR pathway in pancreatic cancer cells, we first established that H_2_O_2_ triggered Chk1 and RPA32 phosphorylation in human pancreatic cancer PANC1 cells and that ATR-specific inhibitor VE-822 prevented H_2_O_2_-induced Chk1 and RPA32 phosphorylation (Figure 1A). Notably, the H_2_O_2_-induced Chk1 phosphorylation and RPA32 phosphorylation were compromised in siRNA-mediated APE2-KD cells compared with control (CTL) siRNA cells (Lane 2 vs. Lane 4, Figure 1B). To validate the phenotype of oxidative stress-induced ATR DDR pathway is due to APE2 reduction, we performed complementation assays by transfecting recombinant plasmid of full-length *Xenopus* APE2 tagged with YFP (YFP-xAPE2) or control plasmid of YFP in APE2-KD PANC1 cells (Lane 5–8, Figure 1B). Due to the sequence difference between *Xenopus* APE2 and human APE2 in the four targeting regions of APE2-siRNA, YFP-xAPE2 cannot be targeted for protein reduction by APE2-siRNA. Using this siRNA-resistant YFP-xAPE2 approach, we showed that YFP-xAPE2 but not YFP rescued the H_2_O_2_-induced Chk1 and RPA32 phosphorylation in APE2-KD PANC1 cells (Lane 4, 6, and 8, Figure 1B). Because anti-human APE2 antibodies do not detect *Xenopus* APE2 protein and anti-*Xenopus* APE2 antibodies do not recognize human APE2 protein, it is a technical difficulty to directly detect and compare endogenous human APE2 and ectopically expressed YFP-xAPE2 via immunoblotting analysis in our rescue experiment. Our control experiment showed that the expression of YFP-xAPE2 and YFP was similar in APE2-KD PANC1 cells regardless of H_2_O_2_ treatment (Figure 1C). These observations suggest that APE2 is critical for the ATR-Chk1 DDR pathway in oxidative stress in PANC1 cells. To exclude the possible cell-specific role of APE2 in the ATR DDR pathway, we performed similar experiments in another human pancreatic cancer MiaPaCa2 cells and found that APE2 was also important for the H_2_O_2_-induced ATR-Chk1 DDR pathway in MiaPaCa2 cells (Supplementary Figures 1A,B). Thus, the above findings demonstrate the important role of APE2 in the ATR-Chk1 DDR pathway following oxidative stress in human pancreatic cancer cells.

To test whether APE2 is a general regulator in the activation of the ATR-Chk1 DDR pathway, we investigated other stressful conditions such as GEM-induced stalled DNA replication forks and CPT/ETO-induced DSBs. Consistent with the ATR DDR pathway by H_2_O_2_-induced oxidative stress, Chk1 and RPA32 phosphorylation was triggered by GEM, CPT, or ETO in PANC1 cells with the treatment of CTL-siRNA but not APE2-siRNA, suggesting that APE2 plays an important role in ATR DDR under various stressful conditions in PANC1 cells (Figure 1D). We also noted similar findings of APE2 in the regulation of the ATR-Chk1 DDR pathway under these different stressful conditions in MiaPaCa2 cells (Supplementary Figure 1C). Our control experiments demonstrated that cell viability under unstressed conditions was reduced ∼25–30% in APE2-siRNA cells compared with CTL-siRNA cells in PANC1 and MiaPaCa2 (Supplementary Figures 1D,E), which is consistent with previous finding that APE2-knockout in mice leads to abnormal cell proliferation and cell cycle progression (Ide et al., 2004). Overall, our observations suggest that APE2 regulates the ATR DDR pathway in response to different stressful conditions in human pancreatic cancer cells.

### APE2-KD by siRNA Leads to Severe DNA Damage and More Micronuclei in Pancreatic Cancer Cells

To determine the role of APE2 in protecting cells from various stressful conditions, we chose to measure γH2AX status in pancreatic cancer cells under normal or damaging environments (e.g., treatment of H_2_O_2_, GEM, CPT, or ETO). Our fluorescence microscopy analysis shows that the percentage of γH2AX-positive cells in APE2-KD PANC1 cells was higher than that in CTL-siRNA PANC1 cells regardless of the treatment of H_2_O_2_ or GEM (Figures 5A,B). We also noted similar observations from the treatment of CPT or ETO (Figure 5C). Similarly, we found that APE2-KD by siRNA led to severe γH2AX under normal conditions or after treatment of H_2_O_2_, CPT, or ETO in MiaPaCa2 cells (Supplementary Figures 2A–D). These observations suggest that APE2 may protect pancreatic cancer cells from DNA damage such as SSBs and DSBs derived from both endogenous and exogenous sources.

A recent study has shown the critical function of APE2 in the regulation of homologous recombination (HR)-mediated DSB repair in MM (Kumar et al., 2018). Micronuclei, a common feature of chromosome instability, are formed due to mitotic errors that mis-segregate intact chromosomes, errors in DNA replication, or repair defects that generate acentric chromosome fragments (Terradas et al., 2010; Guo et al., 2021). To further validate the critical role of APE2 in DSB repair, we examined the micronuclei formation in pancreatic cancer cells under normal or DSB-generating conditions. Our microscopy analysis demonstrated that more percentage of micronuclei-positive cells were observed in APE2-KD PANC1 cells regardless of the treatment of CPT or ETO (Figures 5D–E). We also observed similar results on the role of APE2 in micronuclei formation in MiaPaCa2 cells (Supplementary Figures 2E–F). These observations of severe γH2AX and micronuclei formation in APE2-KD cells suggest the important functions of APE2 in resolving the stressful environments, consistent with its role in DNA repair of DSBs and SSBs (Kumar et al., 2018; Cupello et al., 2019).

### Function of APE2 in the SSB-Induced ATR DDR Pathway Is Compromised by a Distinct APE2 Inhibitor Celastrol in *Xenopus* Egg Extracts

To potentially translate the basic mechanisms of APE2 function in the DDR pathway into future cancer therapy, we sought to identify small molecule inhibitors of APE2 functions. From an unbiased screen of 9,195 compounds, four small-molecule compounds (Dihydrocelastryl, Anthothecol, Erysolin, and MARPIN) were identified to selectively inhibit Chk1 phosphorylation induced by stalled DNA replication forks in p53-deficient cells (Kawasumi et al., 2014). However, the underlying mechanism of how these identified compounds inhibit Chk1 phosphorylation directly or indirectly remains unclear. Dihydrocelastryl is structurally similar to Celastrol, which is a natural compound derived from *thunder god vine* and has been implicated in therapies for cancers such as pancreatic cancer and prostate cancer as an HSP90 modulator and/or proteasome inhibitor (Figure 3A; Hieronymus et al., 2006; Zhang et al., 2008). We recently characterized the requirement of APE2 in the ATR-Chk1 DDR pathway activation in response to defined SSB structures in the *Xenopus* high-speed supernatant (HSS) system (Willis et al., 2012; Cupello et al., 2016; Lin et al., 2018, 2019). First, we intended to test whether Celastrol affects the SSB-induced ATR-Chk1 DDR pathway. As expected, Chk1 phosphorylation was induced by defined SSB plasmid but not control (CTL) plasmid in the *Xenopus* HSS system. Importantly, the SSB-induced Chk1 phosphorylation in the HSS system was compromised by the addition of Celastrol (Figure 3B).

Next, we sought to elucidate how Celastrol regulates the ATR-Chk1 DDR pathway. Due to the significance of the C-terminal Zf-GRF motif of APE2 in the SSB-induced ATR DDR pathway, we tested whether Celastrol affects the binding of APE2 Zf-GRF motif to ssDNA. Our GST-pulldown experiments show that GST-Zf-GRF but not GST associated the beads coupled with ssDNA (Figure 3C), consistent with the APE2 Zf-GRF-ssDNA interaction from previous studies (Wallace et al., 2017; Lin et al., 2018). Notably, the binding of GST-Zf-GRF to ssDNA was compromised by Celastrol (Figure 3C). Furthermore, EMSA assays demonstrated that GST-Zf-GRF but not GST associated with 70-nt ssDNA in a dose-dependent manner *in vitro* (Supplementary Figure 3A), and that such Zf-GRF-ssDNA association was compromised by Celastrol (Figure 3D). To determine the potential role of Celastrol in the regulation of APE2 functions, we turned to the PCNA-mediated APE2 exonuclease activity (Figure 3E; Burkovics et al., 2009; Lin et al., 2018). Notably, Celastrol impaired the 3′-5′ end resection of dsDNA with a gapped structure by recombinant *Xenopu*s APE2 and PCNA in a dose-dependent manner *in vitro* exonuclease assays, suggesting that APE2 3′-5′ exonuclease activity is inhibited by Celastrol (Figure 3E).

To test the specificity of the negative regulation of Celastrol in APE2 Zf-GRF binding to ssDNA and its exonuclease activity, we performed a couple of control experiments. RPA protein complex includes RPA14, RPA32, and RPA70 and has been demonstrated to preferentially associate with ssDNA (Marechal and Zou, 2015; Acevedo et al., 2016). Our EMSA assays showed that Celastrol had almost no effect on the binding of recombinant His-tagged RPA complex to a 70nt-ssDNA (Supplementary Figure 3B). Despite some structure and function similarities, APE1 and APE2 display distinct exonuclease and AP endonuclease activities (Li and Wilson, 2014; Lin et al., 2021). Our EMSA assays demonstrated almost no difference on the association of GST-APE1 with a 70nt-ssDNA by Celastrol in comparison to DMSO (Figure 3F). Furthermore, Celastrol had almost no noticeable effect on the 3′-5′ SSB end resection of dsDNA with a nicked structure by recombinant GST-xAPE1 *in vitro* exonuclease assays (Figure 3G). Overall, our data suggest that Celastrol is a previously uncharacterized small molecule inhibitor of APE2 for its function in SSB end resection and SSB signaling pathway in the *Xenopus* system.

### Celastrol Impairs the ATR-Chk1 DDR Pathway in Pancreatic Cancer Cells

Next, we tested whether Celastrol affects the ATR-Chk1 DDR pathway in human pancreatic cancer cells. First, we found that the binding of recombinant human APE2 protein to ssDNA was also compromised by Celastrol (Figure 4A), suggesting that Celastrol may also affect APE2 functions in human cells. Second, we examined the role of Celastrol for cell viability after 3 days and found that IC_50_ of Celastrol was ∼3.054 and ∼3.032 μM in PANC1 and MiaPaCa2 cells, respectively (Figures 4B,C). Notably, Chk1 phosphorylation and RPA32 phosphorylation induced by H_2_O_2_, GEM, CPT, or ETO were impaired by 1-h pretreatment of Celastrol (2.5 μM) in PANC1 and MiaPaCa2 cells (Figures 4D,E). Our data here support the role of Celastrol in the suppression of the ATR-Chk1 DDR pathway under stress conditions via inhibiting APE2 in human pancreatic cancer cells.

### APE2-KD by siRNA or APE2 Inhibition by Celastrol Sensitizes Pancreatic Cancer Cells to Chemotherapy Drugs

Previous studies show that ATR inhibitor VE-822 sensitizes cancer cells to radiation or chemotherapy drugs such as CPT (Fokas et al., 2012; Josse et al., 2014), and that Chk1-KD by siRNA or Chk1 inhibition by small molecule inhibitor AZD7762 has been shown to function in a synthetically lethal manner with GEM in pancreatic cancers (Venkatesha et al., 2012). Our findings on APE2 in the ATR-Chk1 DDR pathway in both the *Xenopus* system and pancreatic cancer cells prompt us to target the function and regulatory mechanism of APE2 in the ATR DDR pathway for cancer therapy. To directly test whether targeting APE2 functions may sensitize cancer cells to chemotherapy drugs, we took advantage of two strategies developed in this study: APE2 suppression by siRNA-mediated knockdown and APE2 inhibitor Celastrol. Notably, cell viability assays showed that APE2-KD PANC1 cells were more sensitive to H_2_O_2_, GEM, CPT, or ETO than CTL-KD PANC1 cells, suggesting that APE2 suppression sensitizes PANC1 cells to DNA damaging conditions (Figures 2A–D). Similarly, APE2 inhibition by Celastrol also sensitized PANC1 cells to H_2_O_2_-induced oxidative stress and chemotherapy drugs GEM, CPT, and ETO in a dose-dependent manner (0.5, 0.75, and 1 μM) (Figures 2E–H). Similarly, APE2 suppression by siRNA-mediated reduction or Celastrol-mediated inhibition also sensitized MiaPaCa2 cells to oxidative stress or chemotherapy drugs to some extent (Supplementary Figures 4A–H). These observations suggest that pancreatic cancer cells may need APE2-mediated ATR DDR pathway and DNA repair mechanisms to protect from various different stressful conditions including chemotherapy drugs, replication stress or oxidative stress.

## Discussion

### Role of APE2 in the DNA Damage Response

**FIGURE 6:**
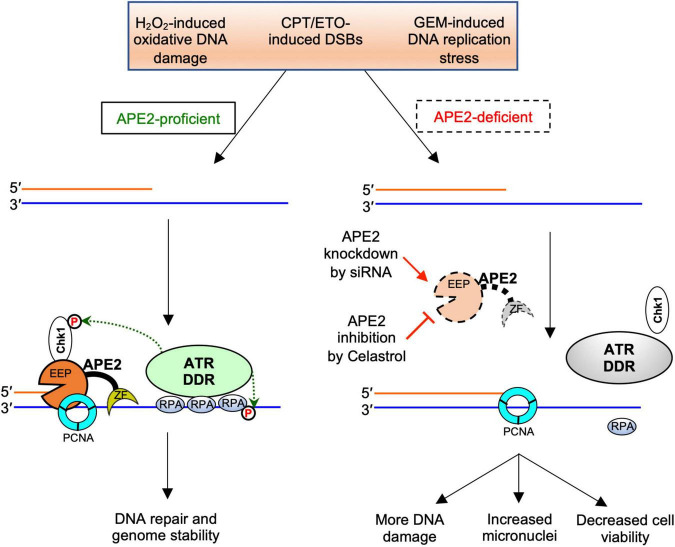
A working model of the general regulator function of APE2 in the ATR-Chk1 DDR pathway. EEP, endonuclease/exonuclease/phosphatase; ZF, Zf-GRF motif; DDR, DNA damage response. See text for more details.

Accumulating evidence suggests that APE2 plays various critical roles in maintaining genome and epigenome integrity (Lin et al., 2021). However, it remains unclear whether APE2 is required for the ATR DDR pathway in mammalian cells. This study demonstrated that APE2 is important for the ATR-Chk1 DDR pathway in response to different stress conditions including oxidative stress, DNA replication stress, and DSBs in pancreatic cancer cells (Figure 1 and Supplementary Figure 1). Fluorescence microscopy analysis shows that APE2-KD by siRNA leads to a higher percentage of γH2AX and more micronuclei under normal or stress conditions in pancreatic cancer cells (Figure 5 and Supplementary Figure 2). Furthermore, we identified a small molecule Celastrol as the first APE2 inhibitor that prevents the binding of APE2 Zf-GRF to ssDNA, APE2’s 3′-5′ exonuclease activity, and the SSB-induced ATR-Chk1 DDR pathway in the *Xenopus* HSS system (Figure 3 and Supplementary Figure 3). Notably, Celastrol treatment impairs the ATR-Chk1 DDR pathway in pancreatic cancer cells (Figure 4). Finally, APE2 suppression by siRNA-mediated knockdown or APE2 inhibition by small molecule inhibitor Celastrol can sensitize pancreatic cancer cells to chemotherapy drugs including GEM, CPT, and ETO (Figure 2 and Supplementary Figure 4). These observations from this study indicate the important role of APE2 in the DNA damage response to maintain genome integrity in mammalian cells. Here, we propose a working model how APE2 especially its exonuclease activity contributes to genome stability in pancreatic cancer cells: (**I**) in APE2-proficient cells, APE2 may process oxidative DNA damage, DSBs, and stalled forks to generate longer region of ssDNA coated with RPA for ATR DDR activation, leading to Chk1 phosphorylation and RPA32 phosphorylation; and (**II**) in APE2-deficient cells, siRNA-mediated APE2-KD or Celastrol-mediated APE2 inhibition (e.g., via ssDNA interaction and exonuclease activity) results in defects of RPA-ssDNA formation and ATR DDR activation, leading to more DNA damage, increased micronuclei, and decreased cell viability (Figure 6).

Previous studies have demonstrated that APE2 is required for the ATR DDR pathway in response to oxidative stress and defined SSB structures in *Xenopus* egg extracts (Willis et al., 2013; Wallace et al., 2017; Lin et al., 2018). Our observations in this study support the important role of APE2 in the ATR-Chk1 DDR pathway in response to hydrogen peroxide-induced oxidative stress, GEM-induced DNA replication stress and CPT/ETO-induced DSBs in human pancreatic cancer cell. These studies collectively support the upstream role of APE2 in the ATR DDR pathway during evolution, although future studies are needed to directly test whether the role of APE2 in the ATR DDR pathway is conserved in other cell types or under other DNA damaging conditions. It is noted that a recent genome-wide CRIPR/Cas9 screen identified APE2 as one of the 117 genes whose mutation leads to hypersensitivity to ATR inhibitors (Hustedt et al., 2019). Although different cell lines and experimental approaches may partially explain the discrepancy with the findings in this study, it is also possible that APE2 contributes to genome integrity via multiple mechanisms in addition to the ATR DDR pathway. Consistent with this speculation, it has been reported that APE2 is important for the HR-mediated DSB repair in MM cells (Kumar et al., 2018). It is interesting to test whether targeting APE2 via small molecule inhibitor such as Celastrol can sensitize cancer cells to ATR inhibitors.

### Role of APE2 in the Maintenance of Genome Integrity

What are the phenotypes of APE2-KD? A prior APE2-knockout (KO) mice study demonstrated that the S and G2/M phases of the cell cycle were significantly increased in APE2-KO thymocytes compared with the wild type (Ide et al., 2004). Similarly, the G2/M phase was arrested in proliferating but not unstimulated APE2-KO splenocytes compared with the wild type (Ide et al., 2004). Consistent with these cell cycle phenotypes of APE2-KO, expression of 74 cell cycle related genes was altered in APE2-KO thymus (Dan et al., 2008). Although exact underlying mechanisms of APE2 in cell cycle regulation need further investigation, an independent group recently reported that APE2 is positively correlated with cell cycle and MYC pathway, and that APE2-KD can suppress CCNB1 and MYC expression likely at the transcription level (Zheng et al., 2020).

Our data demonstrate the critical function of APE2 in the protection of pancreatic cancer cells from DNA damaging conditions (Figure 5). Previous studies have shown that APE2 is critical for the SSB repair pathway in *Xenopus* egg extracts, and HR-mediated DSB repair pathway in MM cells (Kumar et al., 2018; Cupello et al., 2019). A recent CRISPR/Cas9-mediated genetic screen identified APE2 as a synthetic lethal target of BRCA2 in human colon epithelial cell line DLD-1 cells and human ovarian cancer cells PEO1 cells (Mengwasser et al., 2019). Although the underlying mechanism remains unknown, more γH2AX was observed in APE2-knockout (KO) PEO1 cells under unperturbed condition (Mengwasser et al., 2019). Nonetheless, this study is consistent with our observation of increased γH2AX and micronuclei in APE2-KD PANC1 and MiaPaCa2 cells under unperturbed and stress conditions (Figure 5 and Supplementary Figure 2). Furthermore, the function of APE2 in protecting cells from DNA damage and micronuclei under different stress conditions (Figure 5) is in line with its role in SSB repair and DSB repair mechanisms to promote survival in cancer cells. Alternatively, the protection of cancer cells from DNA damaging conditions by APE2 may be mediated from its critical function in the ATR-Chk1 DDR pathway indirectly due to the role of ATR in genome integrity.

### Distinct Role of Celastrol as APE2 Inhibitor

Small molecule inhibitors targeting multi-function protein APE1 in DNA repair and redox signaling (e.g., Methoxyamine, AR03, APE1 inhibitor III, and E3330/APX3330) have been identified and characterized, and E3330/APX3330 as APE1 redox inhibitor has entered and completed Phase I clinical trials in patients with advanced solid tumors (NCT03375086) (Shahda et al., 2019; Caston et al., 2021). However, there is no any specific and/or non-specific small molecule inhibitor targeting APE2 functions from the literature. To the best of our knowledge, Celastrol is the first characterized APE2 inhibitor that impairs APE2’s function in the ATR DDR pathway both in the *Xenopus* system and pancreatic cancer cells via negative regulation of ssDNA binding and catalytic function of APE2.

Our recent studies have shown that APE1 and APE2 as well as their exonuclease but not AP endonuclease activity are important for the SSB-induced Chk1 phosphorylation in the *Xenopus* system (Lin et al., 2018, 2020). Our data in this study demonstrate the inhibitory effect of Celastrol on the ssDNA binding of APE2 Zf-GRF, but not APE1 nor RPA (Figure 3 and Supplementary Figure 3). Furthermore, exonuclease activity of APE2 but not APE1 was compromised by Celastrol, which may explain the suppression of SSB-induced Chk1 phosphorylation by Celastrol in the *Xenopus* system (Figure 3). More importantly, Celastrol treatment can sensitize pancreatic cancer cells to chemotherapy drugs including GEM, CPT, and ETO, which is similar to the phenotype of APE2-KD cells as expected (Figure 2 and Supplementary Figure 4). Although it is not possible to rule out the possibility that other Celastrol targets other than APE2 may also contribute partially to the decreased cell viability, at least the impairment of the ATR DDR pathway via Celastrol-mediated APE2 inhibition is one of the underlying mechanisms.

Although Celastrol exhibits anti-cancer and anti-inflammation activities in previous studies, the translational implication of Celastrol remains limited due to toxicity and narrow therapeutic window as a single agent (Cascao et al., 2017; Chen et al., 2018). Of note, Celastrol (0.5–1 μM) at lower micromolar concentrations than IC_50_ (∼3 μM) can sensitize cancer cells to chemotherapy drugs. Due to the inhibitory effect of Celastrol in the ATR DDR pathway, it will be interesting to test in future studies whether cancer cells with deficiency in ATM or BRCA1/2 are more vulnerable to Celastrol, and whether Celastrol in combination with other small molecules such as PARP1 inhibitors can sensitize cancer cells synergistically. Future follow-up studies are also warranted to identify the possible direct binding site (s) of Celastrol within APE2 and to characterize the APE2 interaction and inhibition by structural approaches. In addition, Celastrol may be further developed and optimized to more specific and efficient APE2 inhibitors in future studies.

### Targeting ATR and Its Regulators in the DNA Damage Response Pathway for Cancer Therapy

Whereas ATR inhibitors with combinations of radiotherapy or chemotherapy have synergistic effects in cancer therapies (Josse et al., 2014; Wallez et al., 2018; Bradbury et al., 2020), regulators/modulators of the ATR-Chk1 DDR pathway have also been targets for cancer therapy. For example, a negative selection RNAi screen from over 10,000 genes in pancreatic cancer BxPC-3 cells identified Rad17, an important regulator of the ATR-Chk1 DDR pathway (Zou et al., 2002; Cimprich and Cortez, 2008), as the most significant synthetic lethal target with GEM treatment, and validation experiments showed that Rad17-KD sensitizes pancreatic cancer cells including BxPC-3, MiaPaCa2, and JoPaca-1 to GEM (Fredebohm et al., 2013). Whereas TopBP1 is a well-established regulator of the ATR-Chk1 DDR pathway (Cimprich and Cortez, 2008; Yan and Michael, 2009), recent studies have demonstrated that TopBP1 promotes prostate cancer progression and that down-regulation of TopBP1 significantly suppressed the proliferation of prostate cancer 22RV1 and LNCaP cells via an apoptosis-mediated mechanism (Li et al., 2020). Rad9-KD via siRNA enhanced sensitivity of breast cancer cell MCF-7 and MDA-MB-231 to doxorubicin that induces DSBs (Yun et al., 2014). Therefore, our findings from this study on the enhanced sensitivity of pancreatic cancer cells to chemotherapy drugs by siRNA-mediated APE2-KD or Celastrol-mediated APE2 inhibition is in with the overall concept that suppressing regulators of the ATR DDR pathway can enhance efficacy of chemotherapies.

### Targeting APE2 in the DNA Damage Response for Future Studies in Cancer Therapy

Does APE2 overexpress in cancer cells compared with normal cells? A pan-cancer analysis from TCGA database and cBioPortal has identified APE2 overexpression at mRNA level in tumor tissues compared with adjacent non-malignant tissues from kidney cancer, breast cancer, lung cancer, liver cancer, and prostate cancer (Jensen et al., 2020). Similarly, APE2 in the MM patient group was overexpressed at mRNA level in comparison to control group monoclonal gammopathy of undetermined significance (MGUS) (Kumar et al., 2018). Furthermore, APE2 overexpression at protein level was also observed in MM cell lines compared with normal cell lines (Kumar et al., 2018). Another independent bioinformatics analysis validated APE2 overexpression in liver cancer and further demonstrated that APE2-high liver cancer patients had a lower overall survival rate compared with APE2-low liver cancer patients regardless of the cancer stages and the hepatitis infection status (Kumar et al., 2018). Thus, APE2 was suggested as an oncogene in liver cancer and could serve as a potential biomarker for cancer screening in the future.

In addition, APE2 was recently identified as a synthetic lethality target in BRCA1/2-deficient cells from a couple of CRISPR-mediated genetic screens, although the exact underlying mechanism remains to be elucidated (Mengwasser et al., 2019; Alvarez-Quilon et al., 2020). Our finding in this study on the critical role of APE2 in the ATR DDR pathway in pancreatic cancer cells provides vital knowledge for future translational studies targeting APE2 functions in various mice models with different genetic backgrounds such as deficiency of ATM or BRCA1/2. Notably, a recent study has shown that chemotherapy drug cisplatin increases APE2 abundance and provokes mitochondrial fragmentation and acute kidney injury (Hu et al., 2021). Thus, targeting APE2 at its expression level or inhibiting its catalytic function via small molecule inhibitor such as Celastrol will provide additional avenues for cancer therapy. While APE2-KD via siRNA or APE2 inhibition via Celastrol sensitizes PANC1 cells or MiaPaCa2 cells to chemotherapy drugs (Figure 2 and Supplementary Figure 4), future investigation is needed to test whether adding back wild type or various mutant hAPE2 to shRNA-mediated APE2-KD or CRISPR/Cas9-mediated APE2-knockout stable cell lines can rescue the phenotype of APE2 deficiency. Anticipated findings from these experiments are expected to elucidate the exact functional domains of APE2 critical for cancer cells’ sensitivity to chemotherapy drugs.

Overall, we have demonstrated the important function of APE2 in the ATR-Chk1 DDR pathway in pancreatic cancer cells, which can be targeted for future combination or synthetic lethality therapies for cancers.

## Data Availability Statement

The original contributions presented in the study are included in the article/Supplementary Material, further inquiries can be directed to the corresponding author/s.

## Ethics Statement

The animal study was reviewed and approved by The Institutional Animal Care and Use Committee (IACUC) at University of North Carolina at Charlotte.

## Author Contributions

MAH, YL, and SY designed the experiments. MAH, YL, GD, JL, AM, JM, HZ, and SY performed the experiments. DT and YN provided the critical reagents. MAH, YL, and SY analyzed the data. MAH and SY wrote the manuscript. MAH, YL, GD, DT, YN, JZ, and SY revised the manuscript with input from all authors.

## Conflict of Interest

The authors declare that the research was conducted in the absence of any commercial or financial relationships that could be construed as a potential conflict of interest.

## Publisher’s Note

All claims expressed in this article are solely those of the authors and do not necessarily represent those of their affiliated organizations, or those of the publisher, the editors and the reviewers. Any product that may be evaluated in this article, or claim that may be made by its manufacturer, is not guaranteed or endorsed by the publisher.

## Supplementary Material

Supplementary Figure 1 Important function of APE2 in ATR-Chk1 DDR pathway in pancreatic cancer cells. **(A)** MiaPaCa2 cells were treated with CTL siRNA or APE2 siRNA for 7 days. Plasmid expressing YFP-xAPE2 or YFP was transfected to APE2-KD MiaPaCa2 cells after 3 days of siRNA-mediated knockdown. After 4-h treatment of H_2_O_2_ (1 mM), total cell lysates were extracted and analyzed via immunoblotting as indicated. **(B)** Fluorescence microscopy analysis shows that the YFP-xAPE2 and YFP was expressed similarly in APE2-KD MiaPaCa2 cells. Scale bar, 100 μm. **(C)** The ATR DDR signaling in cell lysates of MiaPaCa2 cells with control (CTL) or APE2 siRNAs after treatment of various DNA damaging condition was examined via immunoblotting analysis as indicated. Cells were treated with H_2_O_2_ (1 mM), GEM (50 μM), CPT (5 μM), or ETO (50 μM) for 4 h. **(D,E)** Cell viability assays show cell proliferations after 3 days of APE2 siRNA vs. CTL siRNA transfected PANC1 cells **(D)** or MiaPaCa2 cells **(E)**. ^∗^ indicates *p* < 0.05; ^∗∗^ indicates *p* < 0.01, *n* = 4.

Supplementary Figure 2 APE2-KD induces substantially more γH2AX and micronuclei in MiaPaCa2 cells. **(A,C)** Immunofluorescence analysis was performed in MiaPaCa2 cells after CTL siRNA or APE2 siRNA with or without treatment of H_2_O_2_ (625 μM for 5 h), CPT (5 μM for 5 h), or ETO (50 μM for 5 h). DAPI, γH2AX, APE2, and merged images from presentative cells were shown in a slide view. Scale bars, 100 μm. **(B,D)** Percentage of γH2AX-positive cells from **(A)** or **(C)** was quantified in **(B)** and **(D)**, respectively. **(E)** Micronuclei formation of MiaPaCa2 after CTL siRNA or APE2 siRNA with or without 5-h treatment of CPT (5 μM) or ETO (50 μM) was examined via microscopy analysis. Red-dotted circles indicated the micronuclei. Scale bar, 5 μm. **(F)** Percentage of micronuclei-positive MiaPaCa2 cells after treatment of CPT or ETO with CTL or APE2 siRNA from **(E)** was quantified. **(B,D,F)** * indicates *p* < 0.05; ** indicates *p* < 0.01; *** indicates *p* < 0.001.

Supplementary Figure 3 *In vitro* EMSA assays. **(A)** EMSA assays show that the binding of GST-Zf-GRF but not GST can form ssDNA (70nt)-protein complex. **(B)** EMSA assays demonstrate that the recombinant His-tagged RPA complex associated with 70nt-ssDNA and that Celastrol had almost no effect on the association of 70nt-ssDNA and RPA complex.

Supplementary Figure 4 APE2-KD or Celastrol sensitized MiaPaCa2 cells to chemotherapy drugs. **(A–D)** Cell viability assays show that APE2-KD MiaPaCa2 cells were more vulnerable to stress conditions (H_2_O_2_, GEM, CPT, or ETO) compared to CTL siRNA transfected cells. **(E–H)** Cell viability assays demonstrate that Celastrol sensitized MiaPaCa2 cells to H_2_O_2_, GEM, CPT, and ETO.
