# Supplementary figures and images for: APE2 Is a General Regulator of the ATR-Chk1 DNA Damage Response Pathway to Maintain Genome Integrity in Pancreatic Cancer Cells
Source: Front Cell Dev Biol. 2021 Nov 2;9:738502. doi: 10.3389/fcell.2021.738502 (PMC8593216; doi:10.3389/fcell.2021.738502)

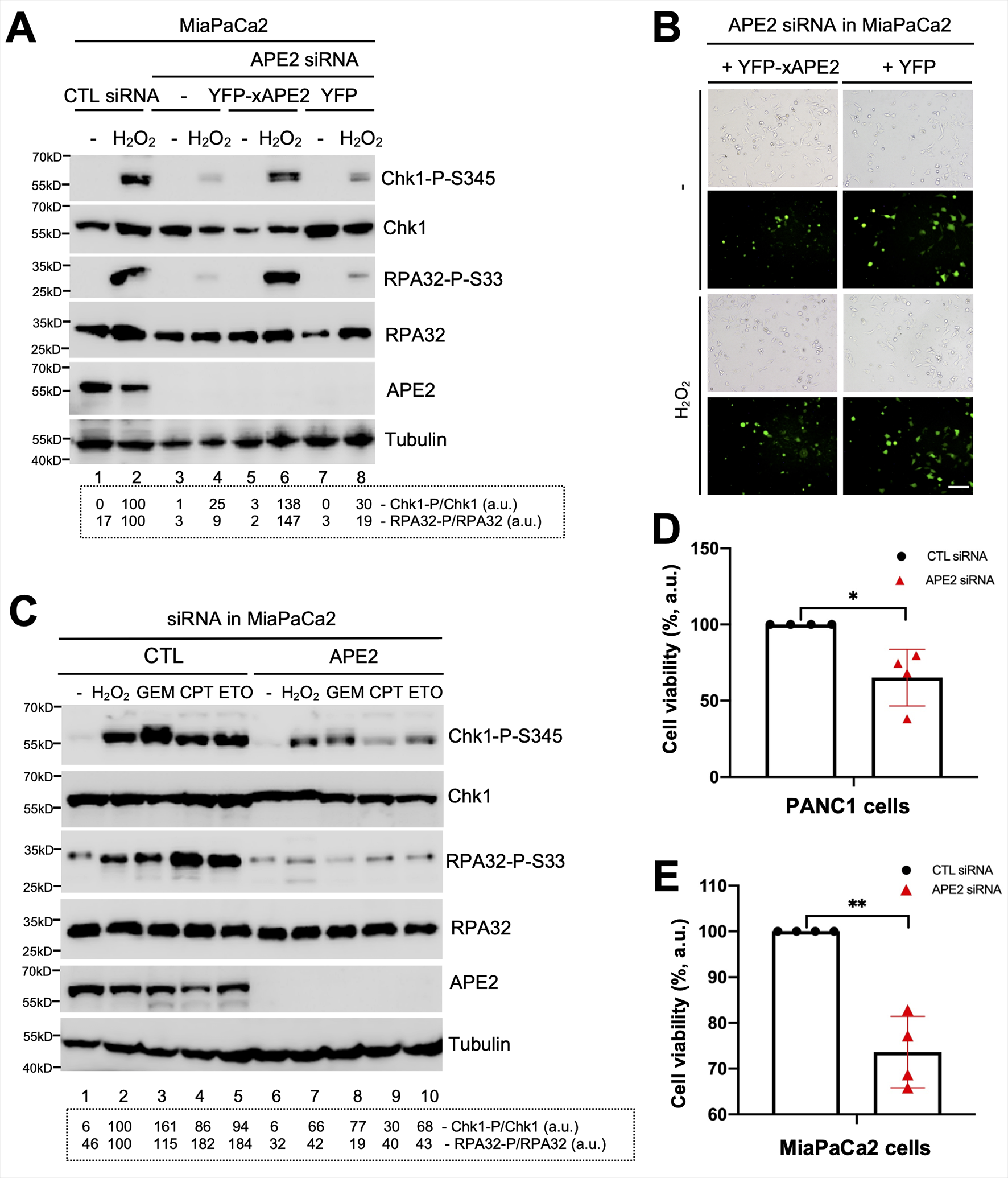

Supplement: Supplementary Figure 1 — Important function of APE2 in ATR-Chk1 DDR pathway in pancreatic cancer cells. (A) MiaPaCa2 cells were treated with CTL siRNA or APE2 siRNA for 7 days. Plasmid expressing YFP-xAPE2 or YFP was transfected to APE2-KD MiaPaCa2 cells after 3 days of siRNA-mediated knockdown. After 4-h treatment of H2O2 (1 mM), total cell lysates were extracted and analyzed via immunoblotting as indicated. (B) Fluorescence microscopy analysis shows that the YFP-xAPE2 and YFP was expressed similarly in APE2-KD MiaPaCa2 cells. Scale bar, 100 μm. (C) The ATR DDR signaling in cell lysates of MiaPaCa2 cells with control (CTL) or APE2 siRNAs after treatment of various DNA damaging condition was examined via immunoblotting analysis as indicated. Cells were treated with H2O2 (1 mM), GEM (50 μM), CPT (5 μM), or ETO (50 μM) for 4 h. (D,E) Cell viability assays show cell proliferations after 3 days of APE2 siRNA vs. CTL siRNA transfected PANC1 cells (D) or MiaPaCa2 cells (E). ∗ indicates p < 0.05; ∗∗ indicates p < 0.01, n = 4. [file Image_1.TIFF]

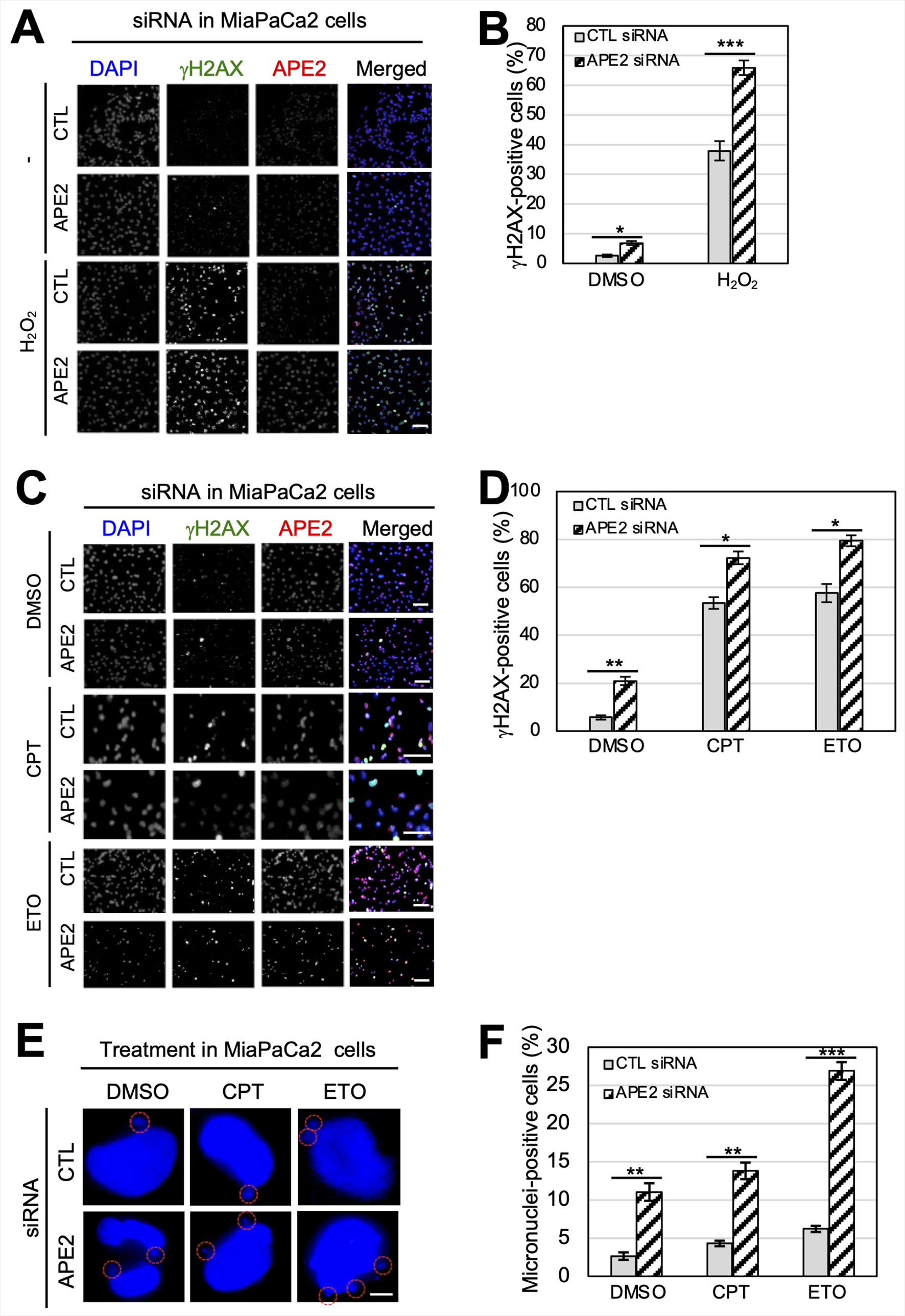

Supplement: Supplementary Figure 2 — APE2-KD induces substantially more γH2AX and micronuclei in MiaPaCa2 cells. (A,C) Immunofluorescence analysis was performed in MiaPaCa2 cells after CTL siRNA or APE2 siRNA with or without treatment of H2O2 (625 μM for 5 h), CPT (5 μM for 5 h), or ETO (50 μM for 5 h). DAPI, γH2AX, APE2, and merged images from presentative cells were shown in a slide view. Scale bars, 100 μm. (B,D) Percentage of γH2AX-positive cells from (A) or (C) was quantified in (B) and (D), respectively. (E) Micronuclei formation of MiaPaCa2 after CTL siRNA or APE2 siRNA with or without 5-h treatment of CPT (5 μM) or ETO (50 μM) was examined via microscopy analysis. Red-dotted circles indicated the micronuclei. Scale bar, 5 μm. (F) Percentage of micronuclei-positive MiaPaCa2 cells after treatment of CPT or ETO with CTL or APE2 siRNA from (E) was quantified. (B,D,F) * indicates p < 0.05; ** indicates p < 0.01; *** indicates p < 0.001. [file Image_2.TIFF]

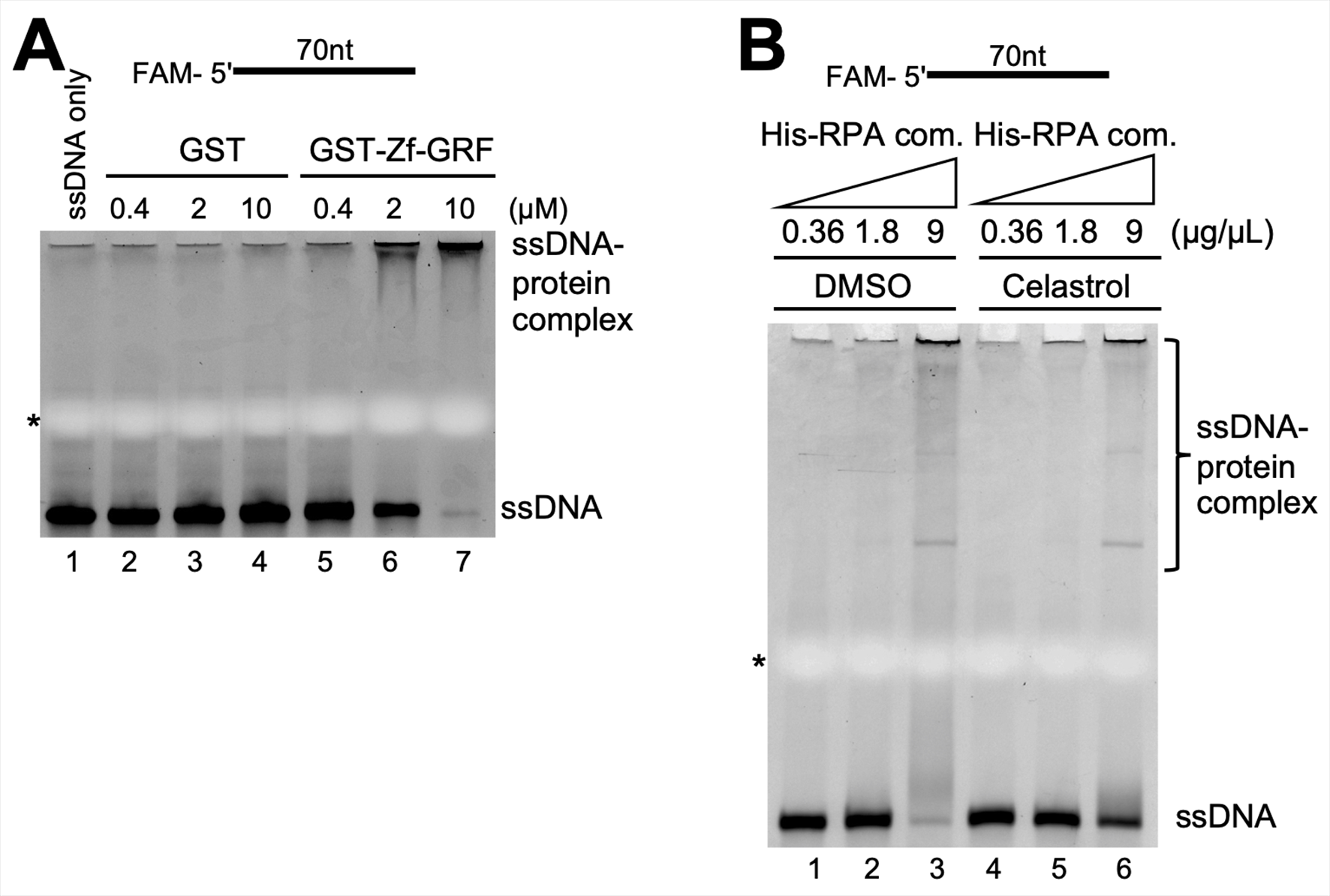

Supplement: Supplementary Figure 3 — In vitro EMSA assays. (A) EMSA assays show that the binding of GST-Zf-GRF but not GST can form ssDNA (70nt)-protein complex. (B) EMSA assays demonstrate that the recombinant His-tagged RPA complex associated with 70nt-ssDNA and that Celastrol had almost no effect on the association of 70nt-ssDNA and RPA complex. [file Image_3.TIFF]

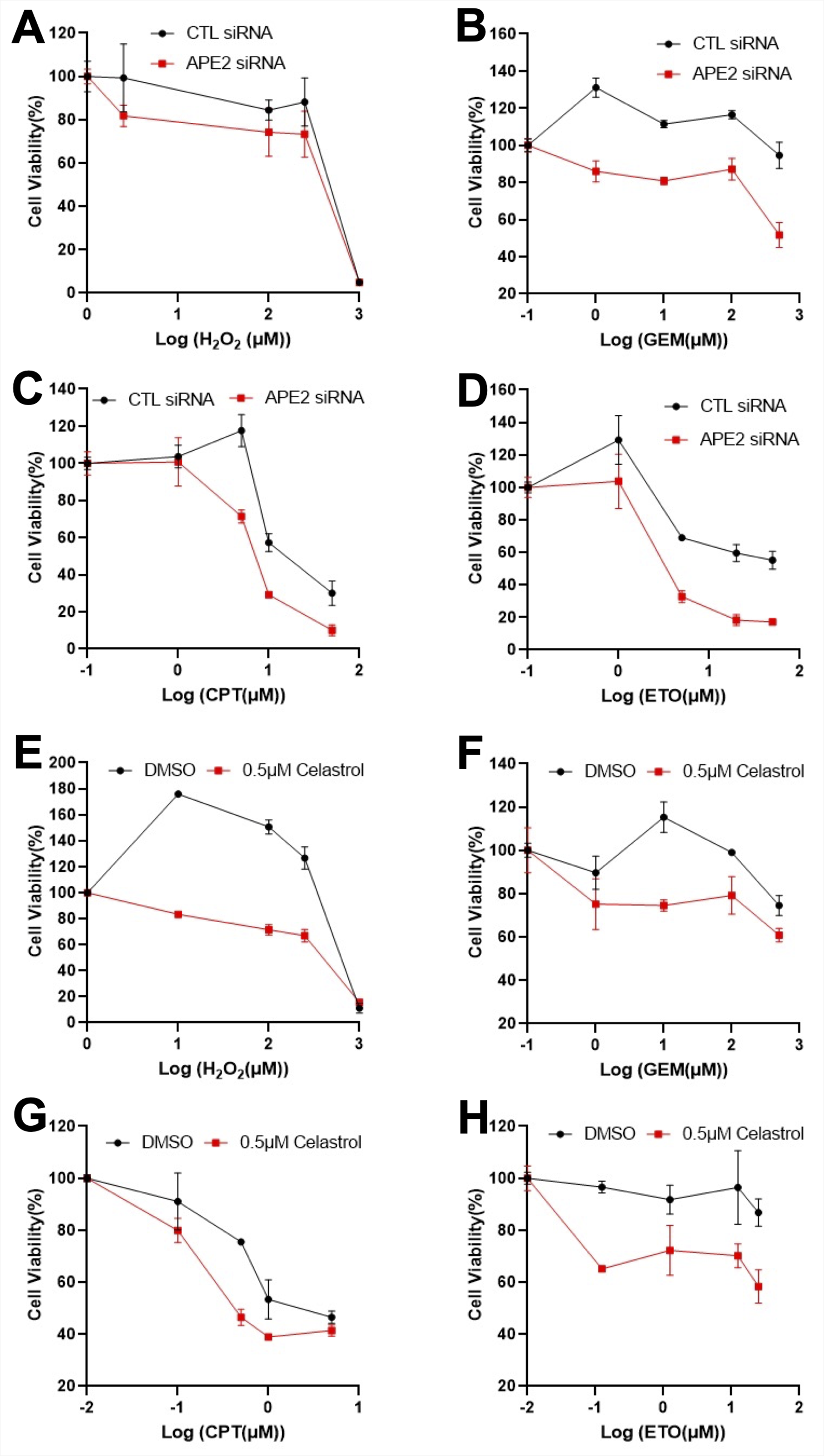

Supplement: Supplementary Figure 4 — APE2-KD or Celastrol sensitized MiaPaCa2 cells to chemotherapy drugs. (A–D) Cell viability assays show that APE2-KD MiaPaCa2 cells were more vulnerable to stress conditions (H2O2, GEM, CPT, or ETO) compared to CTL siRNA transfected cells. (E–H) Cell viability assays demonstrate that Celastrol sensitized MiaPaCa2 cells to H2O2, GEM, CPT, and ETO. [file Image_4.TIFF]
